# Red blood cell distribution width predicts long-term mortality in critically ill patients with acute kidney injury: a retrospective database study

**DOI:** 10.1038/s41598-020-61516-y

**Published:** 2020-03-12

**Authors:** Linpei Jia, Shijun Cui, Jingyan Yang, Qiang Jia, Lixiao Hao, Rufu Jia, Hongliang Zhang

**Affiliations:** 1 Department of Nephrology, Xuanwu Hospital, Capital Medical University, Changchun Street 45#, 100053 Beijing, China; 2 Department of Vascular Surgery, Xuanwu Hospital, Capital Medical University, Changchun Street 45#, 100053 Beijing, China; 3 Central Hospital of Cangzhou, Xinhua Middle Street 201#, 061001 Cangzhou, Hebei Province China; 4 Department of Gerontology, Xuanwu Hospital, Capital Medical University, Changchun Street 45#, 100053 Beijing, China; 5 Department of General Medicine, Xuanwu Hospital, Capital Medical University, Changchun Street 45#, 100053 Beijing, China; 6 Department of Life Sciences, National Natural Science Foundation of China, Shuangqing Road 83#, 100085 Beijing, China

**Keywords:** Acute kidney injury, Risk factors

## Abstract

Acute kidney injury (AKI) is a serious complication in the intensive care unit (ICU), which may increase the mortality of critically ill patients. The red blood cell distribution width (RDW) has proved useful as a predictor of short-term prognosis in critically ill patients with AKI. However, it remains unknown whether RDW has a prognostic value of long-term all-cause mortality in these patients. The data of 18279 critically ill patients with AKI at first-time hospital admission were extracted from the Medical Information Mart for Intensive Care III (MIMIC-III) database. The tertiles of the RDW values were used to divide subjects into three groups, namely RDW < 13.6% for the low RDW group, 13.6% ≤ RDW < 15.2% for the middle RDW group and RDW ≥ 15.2% for the high RDW group. Demographic data, mortality, 4-year survival time and severity scale scores were compared among groups. The Kaplan-Meier analysis and the Cox regression analysis were performed to assess the impact of RDW on all-cause mortality in AKI patients. The receiver operating characteristic (ROC) curve analysis was done to evaluate the prognostic value of RDW on the long-term outcome of critically ill patients with AKI. The median age of the enrolled subjects was 65.6 years. AKI patients with a higher RDW value had significantly shorter survival time and higher death rate. By the Kaplan-Meier analysis, patients in the higher RDW group presented significantly shorter survival time and higher death rate. The Cox regression model indicated RDW as an independent risk factor of all-cause mortality of AKI patients (HR 1.219, 95% CI, 1.211 to 1.228). By the ROC analysis, RDW appeared more efficient in predicting long-term prognosis as compared with conventional severity scales. The AUC of RDW (95% CI, 0.712 to 0.725) was significantly higher than other severity scale scores. In conclusion, RDW is positively correlated to survival time of 4-year follow-up in critically ill patients with AKI, and RDW is an independent prognostic factor of long-term outcomes of these patients.

## Introduction

Acute kidney injury (AKI) is one of the most common complications in critically ill patients, which results in poor prognosis and high risk of death^1^. Approximately 57% of patients in the intensive care unit (ICU) were complicated with AKI, and up to 27% died consequently during hospitalization^2^. When AKI occurs concomitantly with other severe organ dysfunctions like myocardial infarction or sepsis, the mortality rate is further increased to 45% to 60%^3^.

A number of scoring systems are being used to evaluate severity and to predict prognosis of critically ill patients, but none of them is specific for those patients complicated with AKI. Moreover, although various biomarkers are frequently used to predict short-term prognosis and in-hospital mortality of critically ill patients with AKI, including urinary neutrophil gelatinase-associated lipocalin^4^, right ventricular longitudinal strain^5^, microRNAs^6^ and so forth, long-term prognosis is scarcely evaluated due to the difficulty in long-term follows-up. Since AKI may result in permanent injuries of kidney and the long-term risk of death is twice as high in patients with AKI as those without AKI^7^, prediction of long-term prognosis and early intervention for AKI patients are of utmost importance.

The red blood cell distribution width (RDW) represents the variability in size of circulating erythrocytes^8^. Clinically, RDW is useful for the prognostic prediction of acute disease like sepsis^9^ and pancreatitis^10^. Although several studies reported the prognostic efficiency of RDW on AKI, conclusions are inconsistent. Wang *et al*. reported that the short-term prognostic value of RDW was more accurate than Acute Physiology Score III (APS III) and the Sequential Organ Failure Assessment (SOFA) in critically ill patients with AKI^11^. Hu *et al*. also demonstrated that RDW was an independent predictor for AKI and mortality in patients in the coronary care unit^8^. Instead, Elhosseiny *et al*. found that RDW did not correlate with the development of contrast-induced AKI^12^. Besides, all previous studies were focused on the short-term prediction efficiency of RDW on AKI. Whether RDW can be used to predict the long-term prognosis in AKI patients and the efficiency of RDW therein as compared with conventional scales merit further investigation.

In this study, data of critically ill patients complicated with AKI during 48 hours after admission to ICU were extracted. We hypothesized that RDW is useful in the prediction of long-term prognosis of critically ill patients with AKI. We analyzed the relationship between RDW and the survival time of patients at different AKI stages. The prognostic value of 4-year mortality as measured by RDW and/or RDW combined with several severity scales was evaluated.

## Subjects and methods

### Data source

Data were extracted from the Medical Information Mart for Intensive Care III (MIMIC-III) database, which recorded the demographic data, vital signs, medications and other important items of 53,423 adult admissions to ICUs in the Beth Israel Deaconess Medical Center in Boston from 2001 to 2012^13^. The establishment of the MIMIC-III database was approved by the Massachusetts Institute of Technology and the Institutional Review Boards. According to the Health Insurance Portability and Accountability Act (HIPAA) standards (www.hhs.gov), eighteen identifying data elements were removed from the MIMIC-III database^13^. Our study was conducted entirely on publicly available, anonymized data, thus individual patient consents were waived. To get the permission of access to the MIMIC-III database, Linpei Jia passed the Protecting Human Research Participants Exam of National Institutes of Health (Record ID: 27638410).

### Inclusion and exclusion criteria

AKI was defined by the Acute Kidney Injury Network (AKIN) criteria, i.e. serum creatinine (Scr) values increasing ≥ 26.5 μmol/L or 1.5-fold of baseline values during 48 hours, or urine out <0.5 mL/(kg∙h) for more than 6 hours^14^. In our study, we used the AKIN criteria instead of the Kidney Disease: Improving Global Outcomes (KDIGO) criteria for two reasons. One reason is that most data in the MIMIC-III database were recorded before the publication of the KDIGO guideline^15^. Likewise, some other studies used the AKIN criteria for the same reason^16^. The other reason is that during the 7-day follow-up, confounding factors, such as antibiotics and hospital infection, may interfere the kidney function, thus renal function was assessed and AKI was diagnosed within 48 hours after admission^17^. Because Scr values within 3 months before admission were not recorded in the database, the first measurement of Scr within 24 hours after admitted into ICU was set as the baseline^18^.

Included patients were those: (1) with first admission to ICU during hospital stays; (2) with AKI during 48 hours after admitted to ICU; (3) aged ≥18 years old and ≤89 years old; (4) were followed-up for 4 years by the CareVue system^13^. Patients without any RDW data within 24 hours after admission or missing >5% indices were not included in our study. Patients with hospitalization duration longer than 100 days were excluded^19^.

### Data extraction

Data of each patient were extracted from the MIMIC-III database by the Structured Query Language, including age, sex, admission type, ethnicity, marriage, status of renal replacement therapy, RDW values, the survival time, days of hospital stay, and days of ICU stay (Additional File 1). If RDW was measured for several times within 24 hours after the ICU entry, data of the first time were used. Comorbidities were defined as per the Implementation of the International Statistical Classification of Disease and Related Health Problems, 10th Revision (ICD-10) coding systems^20^. APS III^21^, the Modified Logistic Organ Dysfunction System (MLODS)^22^, SOFA^23^, the Oxford Acute Severity of Illness Score (OASIS)^24^ and the Systemic Inflammatory Response Syndrome (SIRS) status^25^ were calculated according to the physiological and laboratory parameters in the MIMIC-III database for estimation of prognosis in AKI patients. All methods were carried out in accordance with relevant guidelines to protect the privacy of patients.

### Grouping

The data distribution of RDW in all the enrolled subjects was calculated; the lower tertile and the upper tertile were 13.6% and 15.2% respectively. We hence divided all of the subjects into three groups based on the tertiles of RDW values^26,27^, namely the low RDW group (RDW < 13.6%), the middle RDW group (13.6% ≤ RDW < 15.2%), and the high RDW group (RDW ≥ 15.2%). Thereafter, we divided patients into subgroups according to AKI stages which are based on the AKIN guidelines^14^. In specific, AKI stage 1 was defined as AKI with an increase of Scr values ≥1.5–1.9 folds or ≥26.5 μmol/L, or urine output <0.5 ml/(kg∙h) for more than 6 hours; AKI stage 2 was defined as AKI with an increase of Scr values ≥2.0–2.9 folds or urine output to <0.5 ml/(kg∙h) for more than 12 hours; AKI stage 3 was defined as AKI with an increase of Scr values ≥ 3.0 folds or ≥354 μmol/L, or urine output <0.3 ml/(kg∙h) for more than 24 hours. Patients with an anuric status >12 hours were also classified as AKI stage 3.

### Outcomes

The MIMIC-III database provides the follow-up data by two systems, namely the Philips CareVue Clinical Information System (models M2331A and M1215A; Philips Health-care, Andover, MA) for four years and the iMDsoft MetaVision ICU (iMDsoft, Needham, MA) for 90 days. Because we aimed at investigating the long-term prognostic effects of RDW, the four-year outcomes after ICU admission in the CareVue system were used. The all-cause death was used as the end-point in our study. We extracted the death status recorded in CareVue system. Since the MIMIC-III database was connected with social security database, which ensures the integrity of follow-up data, we also replenished all-cause death data in four years for patients without recording in the CareVue system.

### Statistics

The trend test of one-way analysis of variance (ANOVA) was used for the analysis of continuous data, and the trend test of Chi-square test was used for categorical data. The linear regression model, the Kaplan-Meier (K-M) curve and the Cox regression model were utilized to analyze the relationship between RDW and survival in AKI patients. The receiver operating characteristic (ROC) curve analysis was performed to compare the area under the ROC curve (AUC), which represented the prognostic efficiency. Statistical analyses were performed with the SPSS 22.0 software (SPSS, IBM, NY, US), GraphPad Prism 7.0 (GraphPad Software, San Diego, US) and the Medcalc 18.5.0 software (MedCalc Software, Ostend, Belgium). Statistical significance was defined as *P* < 0.05. Graphs were generated by Medcalc 18.5.0 and GraphPad Prism 7.0 (GraphPad Software, California, US).

## Results

### Eighteen thousand two hundred and seventy-nine patients were enrolled

**Figure 1:**
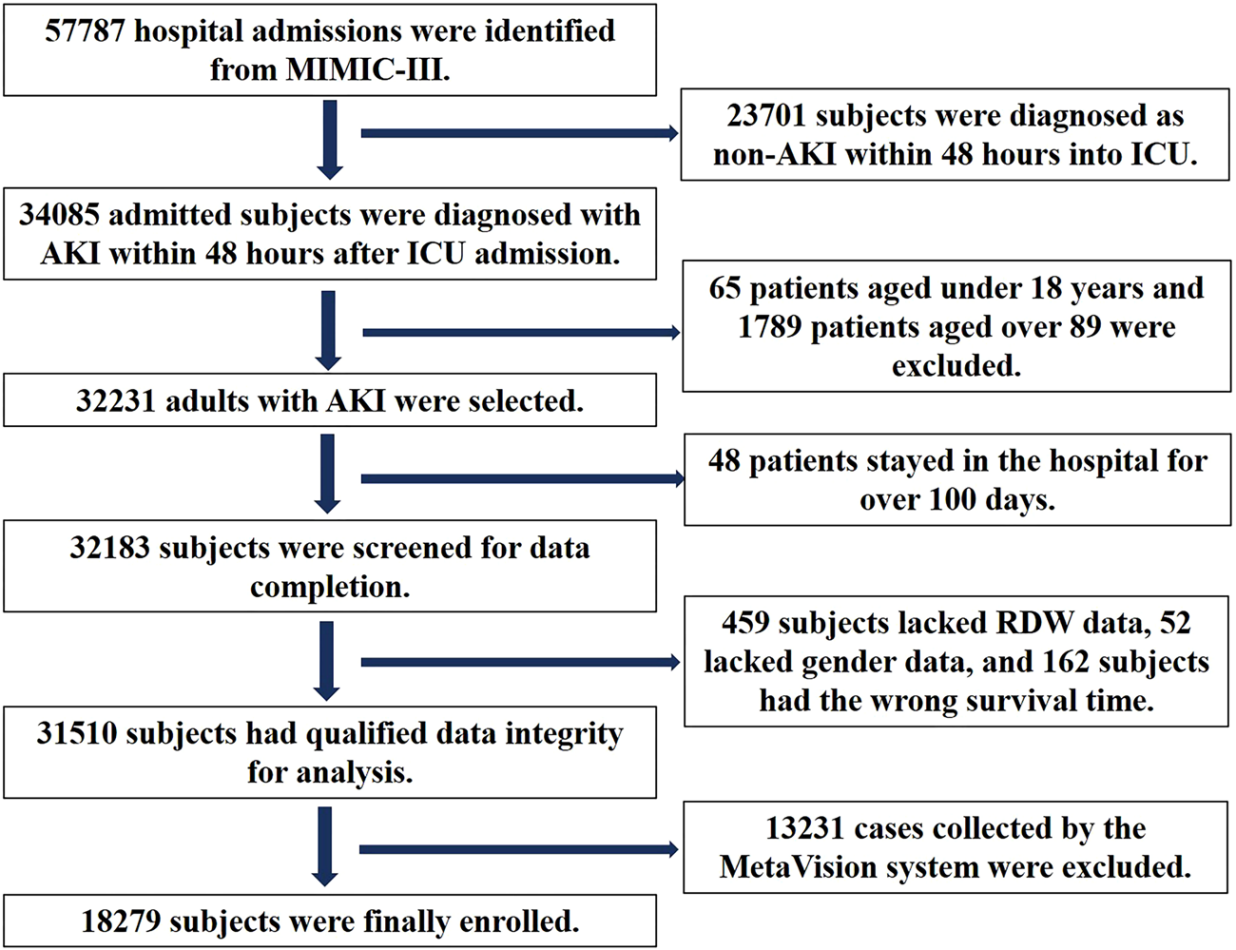
Flowchart of subject screening. Initially, data of 57787 subjects were extracted from the Medical Information Mart for Intensive Care III (MIMIC-III) database. Changes of serum creatinine and urine output within 48 hours after admissions to ICUs were calculated. Then 23701 subjects were excluded as non-acute kidney injury (AKI) patients. Because age of patients over 89 years were marked as 300 or more in the database to protect the privacy, we further excluded 1789 senilities for analysis. After filtering out 65 subjects younger than 18-year-old, 32231 adults were further screened for hospital stays. Hence, 32183 subjects with hospital stay less than 100 days were checked for data integrity, and 459 subjects without red blood cell distribution width (RDW), 52 subjects without sex information and 162 subjects with wrong survival time were excluded. Since the MetaVision system only provided 90-day follow-up data, only 18279 patients with complete follow-up data for 4 years were finally enrolled in our study.

Initially, 57787 subjects were selected from MIMIC-III. After screening, 18279 subjects with mean age of 63.4 ± 16.2 years were enrolled (Fig. 1). The demographic data of the enrolled patients were shown in Table 1. Among these patients, 58.3% were males, and 82.3% were admitted via the emergency room. As the database was constructed in the United States, a majority of the included patients were Caucasian (69.6%). According to the KDIGO guideline, patients at AKI stage 2 accounted for 37.2%, 32.4% of patients were at AKI stage 3, while the others were at AKI stage 1. Only 8.8% of all subjects were treated with renal replacement therapy. Higher severity scale scores represent more severity in critically ill patients with AKI (Table 1). The leading comorbidities were congestive heart failure and chronic pulmonary disease, which accounted for 31.9% and 18.5%, respectively, and 15.9% of the patients were complicated with renal failure. The median hospital stay of all patients was 7.9 days and the median ICU stay was 2.7 days. The all-cause death rate for all subjects was 43.6%.

**Table 1:** Demographic data of study subjects.

	All subjects	Red blood cell distribution width (%)			
		<13.6	13.6–15.2	≥15.2	*P*
		(N = 5696)	(N = 6269)	(N = 6314)	
Age (years)	63.4 ± 16.2	59.6 ± 17.7	65.3 ± 15.4	65.0 ± 14.8	<0.01
Male	10661 (58.3%)	3625 (63.6%)	3612 (57.6%)	3424 (54.2%)	<0.01
Admission type					
Emergency	15050 (82.3%)	4574 (80.3%)	4964 (79.2%)	5512 (87.3%)	<0.01
Urgent	641 (3.5%)	210 (3.7%)	210 (3.3%)	221 (3.5%)	
Elective	2588 (14.2%)	912 (16.0%)	1095 (17.5%)	581 (9.2%)	
Ethnicity					<0.01
Caucasian	12714 (69.6%)	3903 (68.5%)	4466 (71.2%)	4345 (68.8%)	
Black	1765 (9.7%)	334 (5.9%)	534 (8.5%)	897 (14.2%)	
Asian	345 (1.9%)	106 (1.9%)	121 (1.9%)	118(1.9%)	
Others	3455 (18.9%)	1353 (23.8%)	1148 (18.3%)	945 (15.1%)	
Marriage					<0.01
Married	9118 (49.9%)	2893 (50.8%)	3188 (50.9%)	3037 (48.1%)	
Single	4235 (23.2%)	1376 (24.2%)	1354 (21.6%)	1505 (23.8%)	
Others	4926 (26.9%)	1427 (25.1%)	1727 (27.5%)	1772 (28.1%)	
AKI stage					<0.01
1	5557 (30.4%)	1751 (30.8%)	1834 (29.3%)	1970 (31.2%)	
2	6798 (37.2%)	2233 (39.2%)	2529 (40.3%)	2036 (32.2%)	
3	5924 (32.4%)	1710 (30.0%)	1906 (30.4%)	2308 (36.6%)	
Renal replacement therapy					
Yes	1616 (8.8%)	108 (1.9%)	334 (5.3%)	1174 (18.6%)	<0.01
No	16663 (92.4%)	5588 (98.1%)	5935 (94.7%)	5140 (81.4%)	
Severity scale					
APS III	44.71 ± 19.71	37.87 ± 17.07	43.51 ± 18.74	52.07 ± 20.38	<0.01
MLODS	2.89 ± 2.41	2.22 ± 2.18	2.83 ± 2.37	3.57 ± 2.46	<0.01
SOFA	4.52 ± 3.09	3.59 ± 2.61	4.39 ± 2.92	5.51 ± 3.35	<0.01
OASIS	32.08 ± 8.01	30.79 ± 8.47	32.23 ± 8.62	33.09 ± 9.13	<0.01
SIRS	2.85 ± 1.00	2.84 ± 1.02	2.88 ± 0.99	2.82 ± 0.98	<0.01
Comorbidity					
Chronic pulmonary disease	3389 (18.5%)	745 (13.1%)	1265 (20.2%)	1379 (21.8%)	<0.01
Congestive heart failure	5831 (31.9%)	1001 (17.6%)	2069 (33.0%)	2761 (43.7%)	<0.01
Liver disease	1363 (7.5%)	109 (1.9%)	338 (5.4%)	916 (14.5%)	<0.01
Metastatic cancer	924 (5.1%)	125 (2.2%)	269 (4.3%)	530 (8.4%)	<0.01
Renal failure	2898 (15.9%)	216 (3.8%)	738 (11.8%)	1944 (30.8%)	<0.01
Time in hospital (days)	11.3 ± 10.8	9.6 ± 9.3	11.1 ± 10.4	12.9 ± 12.2	<0.01
Time in ICU (days)	5.1 ± 7.2	4.5 ± 6.5	5.1 ± 7.3	5.6 ± 7.6	<0.01
Death	7973 (43.6%)	1314 (23.1%)	2507 (40%)	4152 (65.8%)	<0.01

Note: AKI, acute kidney injury; APS III, acute physiology and chronic health evaluation III; MLODS, modified logistic organ dysfunction system; SOFA, sequential organ failure assessment; OASIS, oxford acute severity of illness score; SIRS, systemic inflammatory response syndrome; ICU, intensive care unit.

### RDW values were negatively correlated with survival time of critically ill patients with AKI

**Figure 2:**
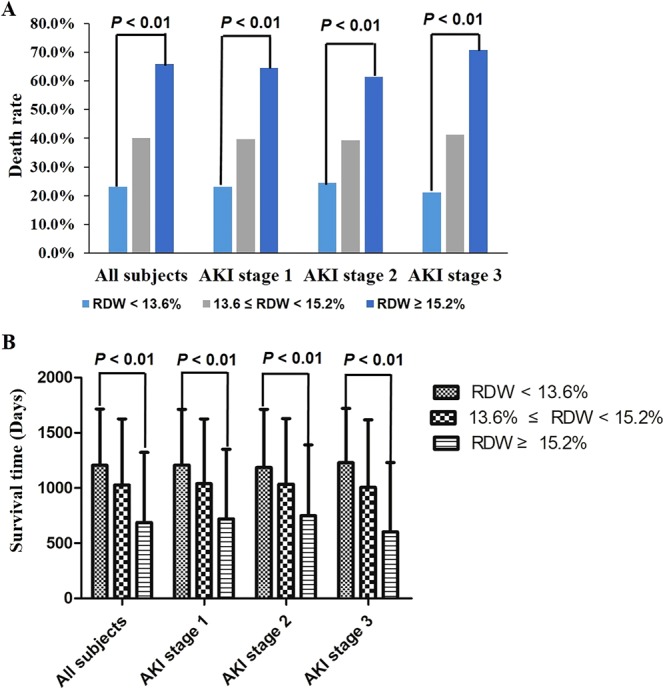
Relationship between red blood cell distribution width (RDW) and all-cause death in critically ill patients with acute kidney injury (AKI). AKI patients were divided into the low (RDW < 13.6%), middle (13.6% ≤ RDW < 15.2%) and high (RDW ≥ 15.2%) RDW groups according to the first RDW measurement. Death rates increased from the low RDW group to the high RDW group for all patients (23.10%, 40.00% and 65.80% for the low, the middle and the high RDW groups respectively, *P* < 0.01, **A**). The same relationship was also found at AKI stage 1 (23.20%, 39.70% and 64.50% for the low, middle and high RDW groups respectively, *P* < 0.01), stage 2 (24.50%, 39.30% and 61.30% for the low, middle and high RDW groups respectively, *P* < 0.01) and stage 3 (21.10%, 41.20% and 70.80% for the low, middle and high RDW groups respectively, *P* < 0.01). With the RDW increasing, survival time decreased from 1205.7 ± 509.5 days to 687.0 ± 635.8 days (*P* < 0.01, **B**). In subgroup analysis, survival time of each RDW groups were also compared at each AKI stage separately, and AKI patients of low RDW group had the longest survival time at all stages (*P* < 0.01, **B**).

Demographic data were compared, and all parameters, including gender, age, admission type, marriage, ethnicity, AKI stage, traditional severity scores, comorbidities, ICU stay and hospital stay, have shown differences among the three RDW groups (all *P* < 0.01, Table 1). In all subjects, the death rate was increased from low to high RDW groups (23.1% for the low RDW group, 40% for the middle RDW group, 65.8% for the high RDW group, Table 1 and Fig. 2A), while the survival time was decreased from low to high RDW groups (Fig. 2B). Similar trends in death rate and survival time were found at each AKI stage (Fig. 2A,B). In the linear regression model, survival time was negatively correlated to RDW values (*r* = 0.35, *P* < 0.01).

### RDW was an independent risk factor of 4-year all-cause mortality in critically ill patients with AKI

**Figure 3:**
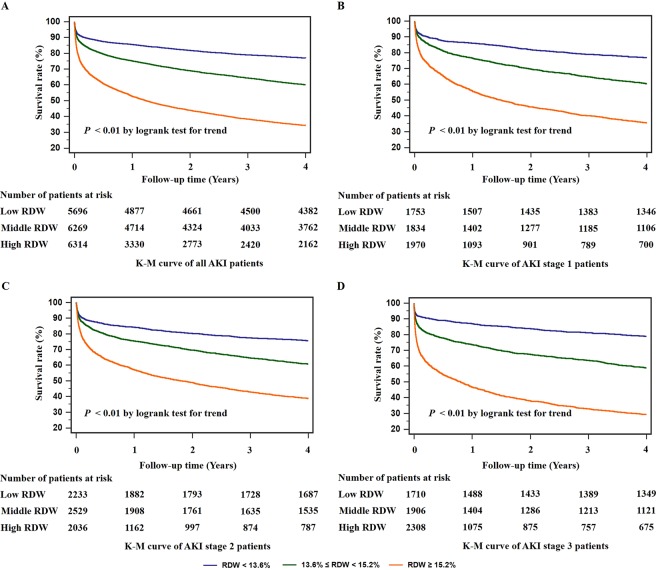
Kaplan-Meier (K-M) survival curves for 4-year all-cause mortalities of critically ill patients with acute kidney injury (AKI). The risk of 4-year overall mortality of each red blood cell distribution width (RDW) group in all participants was shown (**A**). Patients with RDW ≥ 15.2% had the highest all-cause mortality. In subgroup analysis, the same changes of the Kaplan-Meier curves were observed at AKI stage 1 (**B**), stage 2 (**C**) and stage 3 **(D**).

By the K-M analysis, higher RDW values were associated with lower survival rate and shorter survival time (*P* < 0.01 by logrank test for trend, Fig. 3A). We also conducted subgroup analysis of AKI stage 1, stage 2 and stage 3 groups respectively by the K-M curve, while patients with higher RDW values also showed shorter survival time and higher death rate at each stage (*P* < 0.01 by logrank test for trend, Fig. 3B–D). Then the Cox regression models were performed for the association between RDW and all-cause mortality independently as well as adjusted by other severity scale scores. RDW was independently related to the all-cause mortality of AKI patients both in unadjusted and adjusted Cox regression models (*P* < 0.01, Table 2). RDW was also shown as an independent risk factor for all-cause mortality at each AKI stage. In Cox regression models, RDW remained a risk factor of mortality of AKI patients before and after adjustment (Table 3).

**Table 2:** Relationship between RDW and all-cause mortality in Cox model before and after adjustment.

	All subjects (N = 18279)		AKI stage 1 (N = 5557)		AKI stage 2 (N = 6798)		AKI stage 3 (N = 5924)	
	HR (95% CI)	*P*	HR (95% CI)	*P*	HR (95% CI)	*P*	HR (95% CI)	*P*
Unadjusted RDW	1.219 (1.211, 1.228)	<0.01	1.214 (1.197, 1.230)	<0.01	1.202 (1.185, 1.218)	<0.01	1.231 (1.217, 1.245)	<0.01
**RDW adjusted for**								
APS III	1.173 (1.164, 1.182)	<0.01	1.185 (1.168, 1.202)	<0.01	1.172 (1.155, 1.189)	<0.01	1.159 (1.144, 1.174)	<0.01
MLODS	1.194 (1.185, 1.203)	<0.01	1.193 (1.176, 1.210)	<0.01	1.190 (1.174, 1.207)	<0.01	1.185 (1.171, 1.200)	<0.01
SOFA	1.187 (1.178, 1.196)	<0.01	1.189 (1.172, 1.207)	<0.01	1.189 (1.173, 1.206)	<0.01	1.173 (1.158, 1.187)	<0.01
OASIS	1.213 (1.204, 1.222)	<0.01	1.225 (1.208, 1.242)	<0.01	1.199 (1.182, 1.216)	<0.01	1.202 (1.188, 1.217)	<0.01
SIRS	1.222 (1.211, 1.229)	<0.01	1.215 (1.198, 1.232)	<0.01	1.202 (1.186, 1.219)	<0.01	1.228 (1.214, 1.242)	<0.01

Note: AKI, acute kidney injury; HR, hazard ratio; CI, confidence interval; SE, standard error; RDW, red blood cell distribution width; APS III, acute physiology and chronic health evaluation III; MLODS, modified logistic organ dysfunction system; SOFA, sequential organ failure assessment; OASIS, oxford acute severity of illness score; SIRS, systemic inflammatory response syndrome.

**Table 3:** Relationship between RDW and all-cause mortality of patients without chronic kidney disease in Cox model before and after adjustment.

	All subjects (N = 15381)		AKI stage 1 (N = 4520)		AKI stage 2 (N = 6174)		AKI stage 3 (N = 4687)	
	HR (95% CI)	*P*	HR (95% CI)	*P*	HR (95% CI)	*P*	HR (95% CI)	*P*
Unadjusted RDW	1.221 (1.210, 1.231)	<0.01	1.212 (1.192, 1.233)	<0.01	1.206 (1.188, 1.224)	<0.01	1.234 (1.218, 1.250)	<0.01
**RDW adjusted for**								
APS III	1.177 (1.166, 1.188)	<0.01	1.189 (1.168, 1.211)	<0.01	1.177 (1.159, 1.195)	<0.01	1.160 (1.143, 1.178)	<0.01
MLODS	1.202 (1.191, 1.213)	<0.01	1.205 (1.184, 1.226)	<0.01	1.197 (1.179, 1.216)	<0.01	1.190 (1.173, 1.207)	<0.01
SOFA	1.191 (1.181, 1.202)	<0.01	1.199 (1.178, 1.220)	<0.01	1.194 (1.176, 1.212)	<0.01	1.171 (1.154, 1.188)	<0.01
OASIS	1.212 (1.202, 1.223)	<0.01	1.220 (1.200, 1.242)	<0.01	1.201 (1.183, 1.219)	<0.01	1.205 (1.188, 1.222)	<0.01
SIRS	1.220 (1.210, 1.230)	<0.01	1.214 (1.193, 1.234)	<0.01	1.206 (1.188, 1.224)	<0.01	1.230 (1.213, 1.246)	<0.01

Note: AKI, acute kidney injury; HR, hazard ratio; CI, confidence interval; SE, standard error; RDW, red blood cell distribution width; APS III, acute physiology and chronic health evaluation III; MLODS, modified logistic organ dysfunction system; SOFA, sequential organ failure assessment; OASIS, oxford acute severity of illness score; SIRS, systemic inflammatory response syndrome.

### Effects of RDW on 4-year all-cause mortality were not influenced by the history of renal failure in critically ill patients with AKI

**Figure 4:**
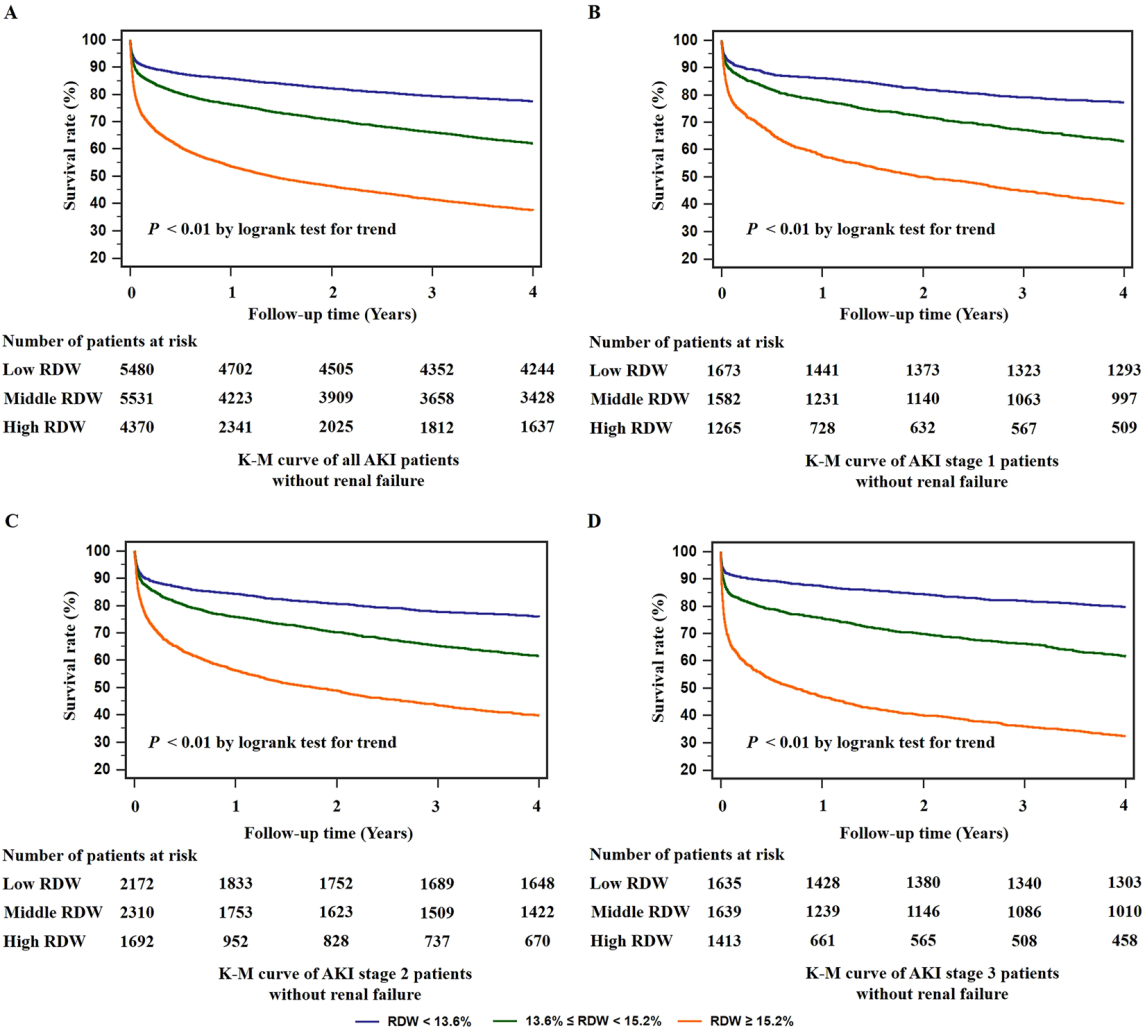
Kaplan-Meier (K-M) survival curves for 4-year overall mortalities of critically ill patients with acute kidney injury (AKI) without comorbidity of renal failure. To eliminate the influences of baseline renal function, we did the Kaplan-Meier analysis for each red blood cell distribution width (RDW) groups in patients without renal failure. The survival rate of the low RDW group was significantly higher than the middle and high RDW groups (*P* < 0.01) for all patients in the 4-year follow-up (**A**). Higher risk of mortality was also shown in the high RDW group at AKI stage 1 (*P* < 0.01, **B**), 2 (*P* < 0.01, **C**) and 3 (*P* < 0.01, **D**) respectively.

Baseline renal function may have an impact on the incidence and mortality of AKI. Hence, we did the survival analysis of RDW on patients without renal failure. The K-M curves indicated that high RDW values were associated with higher all-cause mortality and shorter survival time in all subjects as well as patients at different AKI stages (*P* < 0.01, Fig. 4). Thus, regardless of the baseline renal function, RDW was an independent risk factor for long-term mortality in AKI patients.

### RDW predicted long-term prognosis better than conventional severity scales

**Figure 5:**
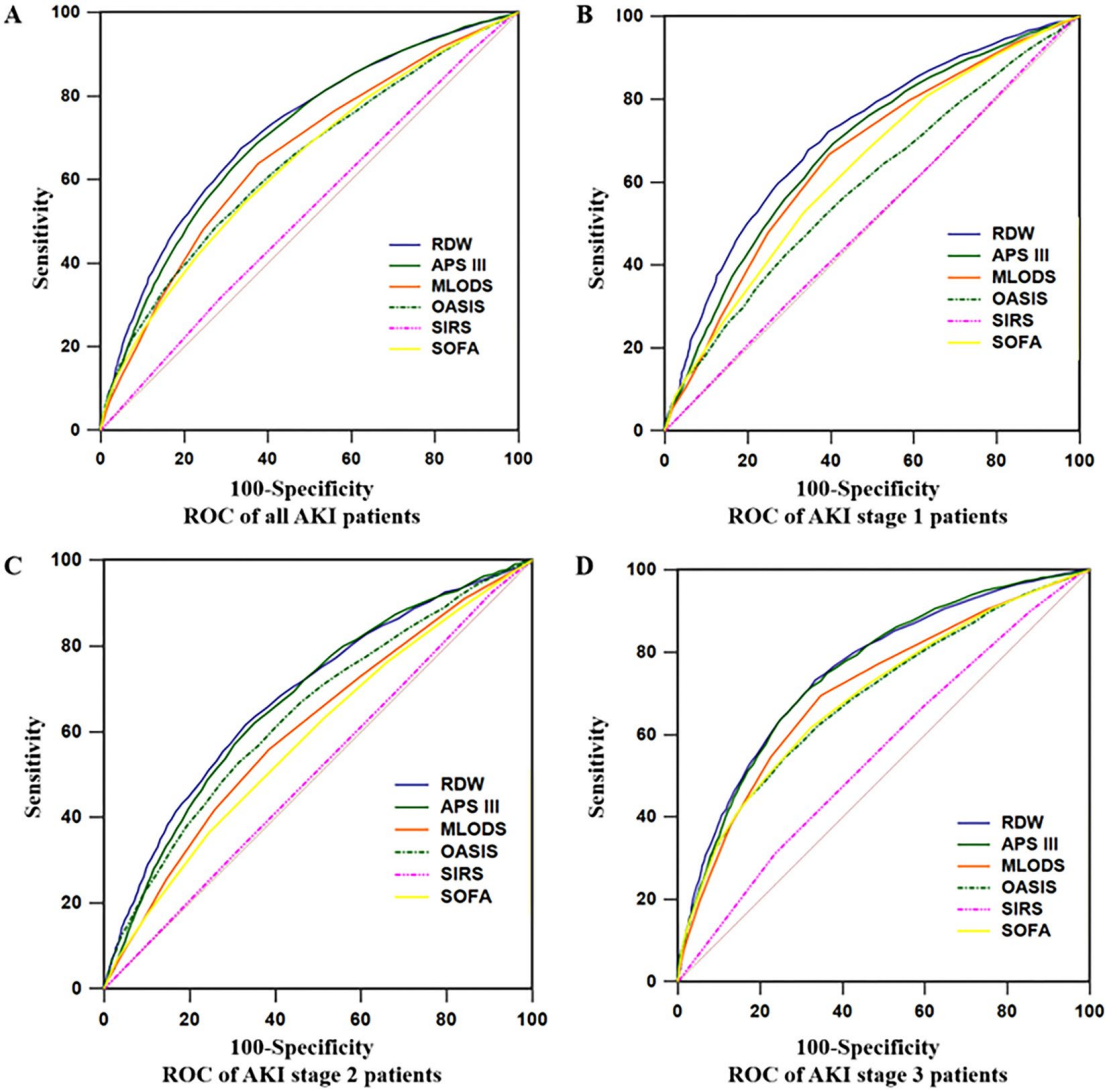
Receiver operating curve (ROC) analyses of predictors of acute kidney injury (AKI) and mortality in intensive care units (ICU) patients. The area under ROC curve (AUC) of red blood cell distribution width (RDW) were compared with Acute Physiology Score III (APS III), the Modified Logistic Organ Dysfunction System (MLODS), the Sequential Organ Failure Assessment (SOFA), the Oxford Acute Severity of Illness Score (OASIS) and the Systemic Inflammatory Response Syndrome (SIRS). RDW values of all subjects (AUC = 0.718, **A**) and AKI stage 1 subjects (AUC = 0.713, **B**) were significantly higher than other severity scale scores (*P* < 0.01). However, at AKI stages 2 and 3, the AUCs of RDW (AUC = 0.684 for stage 2 in **C** and AUC = 0.757 for stage 3 in **D**) showed no difference in comparison with APS III (*P* = 0.297 for stage 2 and *P* = 0.781 for stage 3).

The ROC analysis was performed to evaluate the long-term prognostic value of RDW compared with conventional severity scale scores, including APS III, MLODS, OASIS, SIRS and SOFA. The AUC of RDW for all patients was 0.718 (95% CI, 0.712–0.725), which was significantly higher than other severity scale scores (*P* < 0.01, Fig. 5A and Table 4). For patients at AKI stage 1, RDW with AUC of 0.713 (95% CI, 0.701–0.725) had a better prognostic value than conventional severity scales (*P* < 0.01, Fig. 5B and Table 4). However, for patients with AKI stage 2 and 3, AUC of RDW showed no significant difference from APS III (*P* = 0.297 for AKI stage 2, *P* = 0.781 for AKI stage 3, Fig. 5C,D and Table 4). When RDW was combined with each severity score, AUCs were significantly increased after combination as compared with APS III, MLODS, OASIS, SOFA or RDW alone, respectively (all *P* < 0.01, Figure S1 and Table 4). However, no difference of AUC was found after combination of RDW with SIRS (*P* = 0.080, Figure S1 and Table 4). Taken together, RDW better predicts long-term prognosis of critically ill patients with AKI than traditional severity scoring systems. RDW may also improve the prognostic efficiency of APS III.

**Table 4:** Area under receiver operating curve of RDW and severity scales at different AKI stages.

	All subjects (N = 18279)		AKI stage 1 (N = 5557)		AKI stage 2 (N = 6798)		AKI stage 3 (N = 5924)	
	AUC (95% CI)	*P*	AUC (95% CI)	*P*	AUC (95% CI)	*P*	AUC (95% CI)	*P*
RDW	0.718 (0.712–0.725)		0.713 (0.701–0.725)		0.684 (0.673–0.695)		0.757 (0.746–0.768)	
APS III	0.706 (0.700–0.713)	<0.01	0.682 (0.670–0.695)	<0.01	0.675 (0.664–0.686)	0.297	0.755 (0.744–0.766)	0.781
RDW + APS III	0.758 (0.752–0.764)	<0.01	0.741 (0.729–0.752)	<0.01	0.725 (0.714–0.735)	<0.01	0.802 (0.792–0.812)	<0.01
MLODS	0.659 (0.652–0.666)	<0.01	0.660 (0.647–0.672)	<0.01	0.608 (0.596–0.619)	<0.01	0.709 (0.698–0.721)	<0.01
RDW + MLODS	0.741 (0.735–0.748)	<0.01	0.734 (0.722–0.745)	<0.01	0.700 (0.689–0.711)	<0.01	0.787 (0.776–0.797)	<0.01
SOFA	0.638 (0.631–0.645)	<0.01	0.633 (0.620–0.646)	<0.01	0.586 (0.574–0.597)	<0.01	0.695 (0.683–0.707)	<0.01
RDW + SOFA	0.732 (0.726–0.739)	<0.01	0.724 (0.712–0.736)	<0.01	0.689 (0.678–0.700)	0.115	0.780 (0.769–0.791)	<0.01
OASIS	0.642 (0.635–0.649)	<0.01	0.590 (0.577–0.603)	<0.01	0.643 (0.631–0.654)	<0.01	0.691 (0.679–0.702)	<0.01
RDW + OASIS	0.750 (0.743–0.756)	<0.01	0.726 (0.714–0.738)	<0.01	0.727 (0.717–0.738)	<0.01	0.792 (0.781–0.802)	<0.01
SIRS	0.522 (0.514–0.529)	<0.01	0.506 (0.493–0.519)	<0.01	0.510 (0.498–0.522)	<0.01	0.554 (0.542–0.567)	<0.01
RDW + SIRS	0.720 (0.713–0.726)	0.080	0.713 (0.701–0.725)	0.964	0.685 (0.674–0.696)	0.402	0.760 (0.749–0.771)	0.018

Note: AKI, acute kidney injury; AUC, area under curve; CI, confidence interval; SE, standard error; RDW, red blood cell distribution width; APS III, acute physiology and chronic health evaluation III; MLODS, modified logistic organ dysfunction system; SOFA, sequential organ failure assessment; OASIS, Oxford acute severity of illness score; SIRS, systemic inflammatory response syndrome.

## Discussion

Clinically, RDW is a common parameter for evaluation of anemia and inflammation. In recent years, much attention has been paid to the relationship between renal function and RDW. In this study, we explored the association between RDW and long-term outcomes of 18279 critically ill patients with AKI. Higher levels of RDW predicted shorter survival time and higher long-term death rate. Our findings suggest that RDW might be a potential predictor of all-cause mortality of critically ill patients with AKI.

First, we explored the association between RDW and survival time. We demonstrated that higher RDW predicted worse prognosis of AKI patients. Our results were consistent with previous studies on short-term outcomes, which mainly focused on how RDW was related with survival in AKI patients after percutaneous transluminal coronary intervention or with other coronary diseases, especially contrast-induced AKI^8,28,29^. Survivors of AKI had a lower RDW value. Similar findings about the association between low RDW and high survival rate were also reported in sepsis-induced^30^ and extracorporeal membrane oxygenation treated patients with AKI. Different from previous studies, however, we emphasized that the baseline RDW values also determined the long-term death rate and survival time in multiple-cause AKI.

To adjust for the confounding of baseline renal function, we further examined the relationship between RDW and long-term all-cause mortalities of AKI patients without comorbidity of renal failure. Elevation of blood RDW levels has been used to predict mortalities in chronic kidney disease (CKD) patients both with and without hemodialysis^31,32^. For patients with normal baseline renal function, RDW was positively correlated to 4-year survival rates, which was consistent with Odutayo’s results^33^. Hence RDW may serve as an independent factor of long-term outcomes of critically ill patients with AKI.

We also compared the prognostic value of RDW with traditional severity scores. Oh’s team analyzed the data of 470 renal replacement therapy-treated patients with AKI and found that SOFA with the AUC of 0.694 predicted more accurately than RDW with the AUC of 0.586^34^. Mizuno and colleagues showed the potential predictive ability of RDW combined with the Mehran risk score but not RDW only for contrast-induced AKI in myocardial infarction patients^35^. Acute physiology and chronic health evaluation II scores were also confirmed to be better than RDW in predicting in-hospital mortality as well as 2-year mortality^8^. Inconsistent with previous studies, however, RDW showed a better predictive value of four-year mortality than commonly used severity scoring systems in our study. This inconsistency might be due to differences in sample size and selection of subjects. On one hand, previous studies usually enrolled no more than 1000 subjects, which is far less than the sample size in our study. A large sample could make the results more precise, stable and reliable^36^. On the other hand, subjects in the present study were critically ill patients with AKI caused by various reasons. Heterogeneous causes of AKI might also influence the prognostic efficiency of RDW. Likewise, different factors that may influence short-term and long-term outcomes should be addressed as well^37^. Although traditional severity scales are used mainly to evaluate disease severity and short-term prognosis of acute diseases, studies have proved their usefulness in the long-term prognosis prediction. For example, Pekkarinen’s study indicated that SOFA was associated with 1-year outcome and healthcare costs of patients with cardiac arrest^38^. Hagen’s team found that SIRS could predict poor long-term functional outcome after intracerebral hemorrhage^39^. In line with these findings, we also showed that either RDW or severity scores could be used to predict the long-term prognosis of AKI patients; RDW appeared to be even more efficient.

Although the predictive value of RDW on all-cause mortalities has been revealed, the mechanism remains unknown. Both inflammation^40^ and oxidative stress^41^ may play crucial roles. When inflammation occurs, iron metabolism and bone marrow function are inhibited, and the proliferation and maturation of erythrocytes are inhibited^40^, leading to an increase of RDW values. Meanwhile, the severity of AKI is related to systematic and intrarenal inflammation^42^. SIRS mainly represents an inflammation status of whole body, and our results revealed that SIRS values were increased in the high RDW group. Therefore, RDW could predict the prognosis of AKI partially by reflecting the levels of inflammation. AKI patients in ICU are often complicated with activation of oxidative stress including disturbed metabolism, sepsis, and hemodynamic dysregulations^43^. Oxidative stress plays key roles in the erythroid cell cycle, differentiation and maturation, and RDW has been found associated with various oxidative stress biomarkers, such as serum malondialdehyde and tumor necrosis factor-alpha, in critically ill patients^44^. Usually, the multi-organ dysfunction is accompanied with severe oxidative stress. Hence, elevations of MLODS and SOFA were shown in the high RDW groups.

The large sample size is the advantage of our study. The complete follow-up data make it possible to investigate the long-term prognosis, which is difficult to be observed in cohort studies. However, limitations should be acknowledged. First, as mentioned in the Subjects and methods section, the AKIN criteria instead of the KDIGO criteria was used due to the intrinsic drawback of the MIMIC-III database. Second, some important information was not recorded in the MIMIC-III database, including Scr during the previous 3 months and the past medical history of end stage renal disease (ESRD), and hence estimation of previousrenal function and exclusion of patients with ESRD were impossible. Third, given the missing items of past medical histories in the MIMIC-III database, patients with hematological diseases or renal anemia were difficult to distinguish and select. Hence, effects of hematological diseases on the prognostic values of RDW were not evaluated. Besides, the missing data of some other RDW-influencing pre-existing conditions, such as serious infections, autoimmune diseases and so forth, also made stratified analyses impossible. Fourth, because the major adverse events after discharge were not followed or recorded, the predictive value of RDW on cardiovascular and cerebrovascular events was not evaluated as well.

Further improvement in the MIMIC-III database may make up for the above-mentioned limitations. Regular follows-up for up to 3 to 6 years are necessary. Detailed past medical history, medication history and the exact cause of death should be recorded, which are helpful to explore the prognostic values of RDW on important complications in critically ill patients with AKI.

## Conclusion

Based on the clinical data of 18279 AKI patients in MIMIC-III database, a negative correlation was found between RDW and all-cause mortality. RDW proved to be a predictive parameter of long-term prognosis of critically ill patients with AKI.

## Supplementary information

Supplementary Info.

## Data Availability

All data and material were available at https://mimic.mit.edu/.
