# Supplementary material for: Red blood cell distribution width predicts long-term mortality in critically ill patients with acute kidney injury: a retrospective database study
Source: Sci Rep. 2020 Mar 12;10:4563. doi: 10.1038/s41598-020-61516-y (PMC7067822; doi:10.1038/s41598-020-61516-y)
Supplement: Supplementary file 1 — Supplementary Info. [file 41598_2020_61516_MOESM1_ESM.pdf]

Red blood cell distribution width predicts long-term mortality in critically ill patients with acute kidney injury: a retrospective database study.

Linpei Jia, MD, PhD[1#*], Shijun Cui, MD[2#], Jingyan Yang, MD[3], Qiang Jia, MD[1], Lixiao Hao, MD[4, 5], Rufu Jia, MD[3*], Hongliang Zhang, MD, PhD[6*]

[1]Department of Nephrology, Xuanwu Hospital, Capital Medical University, Changchun Street 45#, 100053, Beijing, China;

[2]Department of Vascular Surgery, Xuanwu Hospital, Capital Medical University, Changchun Street 45#, 100053, Beijing, China;

[3]Central Hospital of Cangzhou, Xinhua Middle Street 201#, 061001, Cangzhou, Hebei Province, China;

[4]Department of Gerontology, Xuanwu Hospital, Capital Medical University, Changchun Street 45#, 100053, Beijing, China;

[5]Department of General Medicine, Xuanwu Hospital, Capital Medical University, Changchun Street 45#, 100053, Beijing, China;

[6]Department of Life Sciences, the National Natural Science Foundation of China, Shuangqing Road 83#, 100085, Beijing, China.

[#]Linpei Jia and Shijun Cui contributed equally as first authors.

*Correspondence and reprint requests should be addressed to: Dr. Linpei Jia, Department of Nephrology, Xuanwu Hospital, Capital Medical University, Changchun Street 45#, 100053, Beijing, China, Email: anny_069@163.com; Dr. Rufu Jia, Central Hospital of Cangzhou, Xinhua Middle Street 201#, 061001, Cangzhou, Hebei Province, China, Email: zxyy5688@126.com; or Dr. Hongliang Zhang, Department of Life

Sciences, National Natural Science Foundation of China, Shuangqing Road 83#, 100085, Beijing, China, Email: drzhl@hotmail.com

Additional file 1: Structured Query Language (SQL) code for searching in the Medical Information Mart for Intensive Care III (MIMIC-III) database.

1) The SQL code for diagnosis and stage of acute kidney injury (AKI) were provided by *mimic-code* in website of github (https://github.com/MIT-LCP/mimic-code/tree/master/concepts/organfailure).

2) The SQL code for calculating Acute Physiology Score III, the Modified Logistic Organ Dysfunction System, Sequential Organ Failure Assessment, the Oxford Acute Severity of Illness Score and the Systemic Inflammatory Response Syndrome status were provided by *mimic-code* in website of github (https://github.com/MIT-LCP/mimic-code/tree/master/concepts/severityscores).

3) The SQL code for demographic data were provided by *mimic-code* in website of github (https://github.com/MIT-LCP/mimic-code/tree/master/concepts/demographics).

4) The SQL code for comorbidities were provided by *mimic-code* in website of github (https://github.com/MIT-LCP/mimic-code/commit/faa276ae7149f81c897103256e23c8282f6d3b99).

5) The SQL codes for data extraction are as following:

```
1# DROP MATERIALIZED VIEW IF EXISTS RDW_24h CASCADE;
CREATE MATERIALIZED VIEW RDW_24h AS
SELECT
        l.hadm_id, value, valueuom, charttime
    FROM labevents l
    inner join admissions ad
        on l.hadm_id = ad.hadm_id
        and l.charttime <= ad.admittime + interval '1' day
        and l.charttime > ad.admittime - interval '1' day
    WHERE l.valuenum IS NOT NULL
    AND l.itemid= 51277
    AND l.valuenum != 0
```

```
2# DROP MATERIALIZED VIEW IF EXISTS rdw_24hfir CASCADE;
CREATE MATERIALIZED VIEW rdw_24hfir AS
select * from (select hadm_id, value, valueuom, charttime, ROW_NUMBER() over(partition by hadm_id order by charttime) as row_sort from rdw_24h )
  as t where t.row_sort=1
3# DROP MATERIALIZED VIEW IF EXISTS AKI_subid CASCADE;
CREATE MATERIALIZED VIEW AKI_subid AS
SELECT kdigo_stages_48hr.icustay_id, icustays.subject_id, icustays.hadm_id, kdigo_stages_48hr.aki_48hr, kdigo_stages_48hr.aki_stage_48hr, kdigo_stages_48hr.aki_stage_48hr_creat, kdigo_stages_48hr.aki_stage_48hr_uo, kdigo_stages_48hr.lowcreat48hr, kdigo_stages_48hr.highcreat48hr, kdigo_stages_48hr.uo24_48hr
FROM kdigo_stages_48hr LEFT OUTER JOIN icustays
ON kdigo_stages_48hr.icustay_id = icustays.icustay_id
4# DROP MATERIALIZED VIEW IF EXISTS AKI_adm CASCADE;
CREATE MATERIALIZED VIEW AKI_adm AS
SELECT aki_subid.*, admissions.ethnicity, admissions.hospital_expire_flag, admissions.insurance, admissions."language", admissions.marital_status, admissions.religion
From aki_subid LEFT OUTER JOIN admissions
ON aki_subid.hadm_id = admissions.hadm_id
5# DROP MATERIALIZED VIEW IF EXISTS AKI_adm2 CASCADE;
CREATE MATERIALIZED VIEW AKI_adm2 AS
SELECT aki_adm.*, heightweight.height_first, heightweight.weight_first, icustay_detail.admission_age, icustay_detail.admission_type, icustay_detail.gender, icustay_detail.admittime, icustay_detail.dischtime, icustay_detail.first_hosp_stay, icustay_detail.first_icu_stay, icustay_detail.hospstay_seq, icustay_detail.icustay_seq, icustay_detail.intime, icustay_detail.outtime, icustay_detail.los_hospital, icustay_detail.los_icu
FROM aki_adm
LEFT OUTER JOIN heightweight ON aki_adm.icustay_id = heightweight.icustay_id
LEFT OUTER JOIN icustay_detail ON aki_adm.icustay_id = icustay_detail.icustay_id
6# DROP MATERIALIZED VIEW IF EXISTS AKI_adm3 CASCADE;
CREATE MATERIALIZED VIEW AKI_adm3 AS
SELECT aki_adm2.*, patients.dob, patients.dod, patients.dod_hosp, patients.dod_ssn, patients.expire_flag, rrt.rrt, icustays.dbsource
FROM aki_adm2 LEFT JOIN patients on aki_adm2.subject_id = patients.subject_id
                        LEFT JOIN rrt on aki_adm2.icustay_id = rrt.icustay_id
                         LEFT JOIN icustays on aki_adm2.icustay_id = icustays.icustay_id
7# DROP MATERIALIZED VIEW IF EXISTS aki_rdw CASCADE;
CREATE MATERIALIZED VIEW aki_rdw AS
SELECT aki_adm3.*,
            rdw_24hfir.value as RDW,
```

```
        sofa.sofa as SOFA,
        apsiii.apsiii as APS3,
        sapsii.sapsii as sapsii,
        oasis.oasis as oasis,
        sirs.sirs as sirs,
        mlods.mlods as mlods,
        nodrgicd.chronic_pulmonary,
        nodrgicd.congestive_heart_failure,
        nodrgicd.liver_disease,
        nodrgicd.metastatic_cancer,
        nodrgicd.renal_failure
FROM aki_adm3 LEFT JOIN nodrgicd ON aki_adm3.hadm_id=nodrgicd.hadm_id
    LEFT JOIN apsiii on aki_adm3.icustay_id=apsiii.icustay_id
    LEFT JOIN sofa on aki_adm3.icustay_id=sofa.icustay_id
    LEFT JOIN sapsii on aki_adm3.icustay_id=sapsii.icustay_id
    LEFT JOIN oasis on aki_adm3.icustay_id=oasis.icustay_id
    LEFT JOIN sirs on aki_adm3.icustay_id=sirs.icustay_id
    LEFT JOIN mlods on aki_adm3.icustay_id=mlods.icustay_id
    LEFT JOIN rdw_24hfir on aki_adm3.hadm_id=rdw_24hfir.hadm_id
```

```
8# DROP MATERIALIZED VIEW IF EXISTS aki_rdw_hadm CASCADE;
CREATE MATERIALIZED VIEW aki_rdw_hadm AS
select * from (select *, ROW_NUMBER() over(partition by hadm_id order by admittime) as row_sort from aki_rdw )
  as t where t.row_sort=1
```

```
9# DROP MATERIALIZED VIEW IF EXISTS aki_rdw_final CASCADE;
CREATE MATERIALIZED VIEW aki_rdw_final as

SELECT *
, ROUND( (CAST(EXTRACT(epoch FROM dod - intime)/(60*60*24) AS numeric)), 4) AS deathtime
, ROUND( (CAST(EXTRACT(epoch FROM dod_hosp - intime)/(60*60*24) AS numeric)), 4) AS deathtime_hosp
, ROUND( (CAST(EXTRACT(epoch FROM dod_ssn - intime)/(60*60*24) AS numeric)), 4) AS deathtime_ssn
FROM aki_rdw_hadm
```

Additional file 2: Figure S1. Receiver operating curve (ROC) analyses of combined use of predictors in intensive care units (ICU) patients with acute kidney injury (AKI). The area under the ROC curves (AUCs) of combination of red blood cell distribution width (RDW) with Acute Physiology Score III (APS III), the Modified Logistic Organ Dysfunction System (MLODS), the Sequential Organ Failure Assessment (SOFA), the Oxford Acute Severity of Illness Score (OASIS) and the Systemic Inflammatory Response Syndrome (SIRS) were compared with RDW and each severity score alone. The AUCs of RDW and each severity score were higher than RDW and each severity score along ($P < 0.01$, **A** for APS III, **B** for MLODS, **C** for SOFA, **D** for OASIS) except for SIRS ($P = 0.080$, **E**).

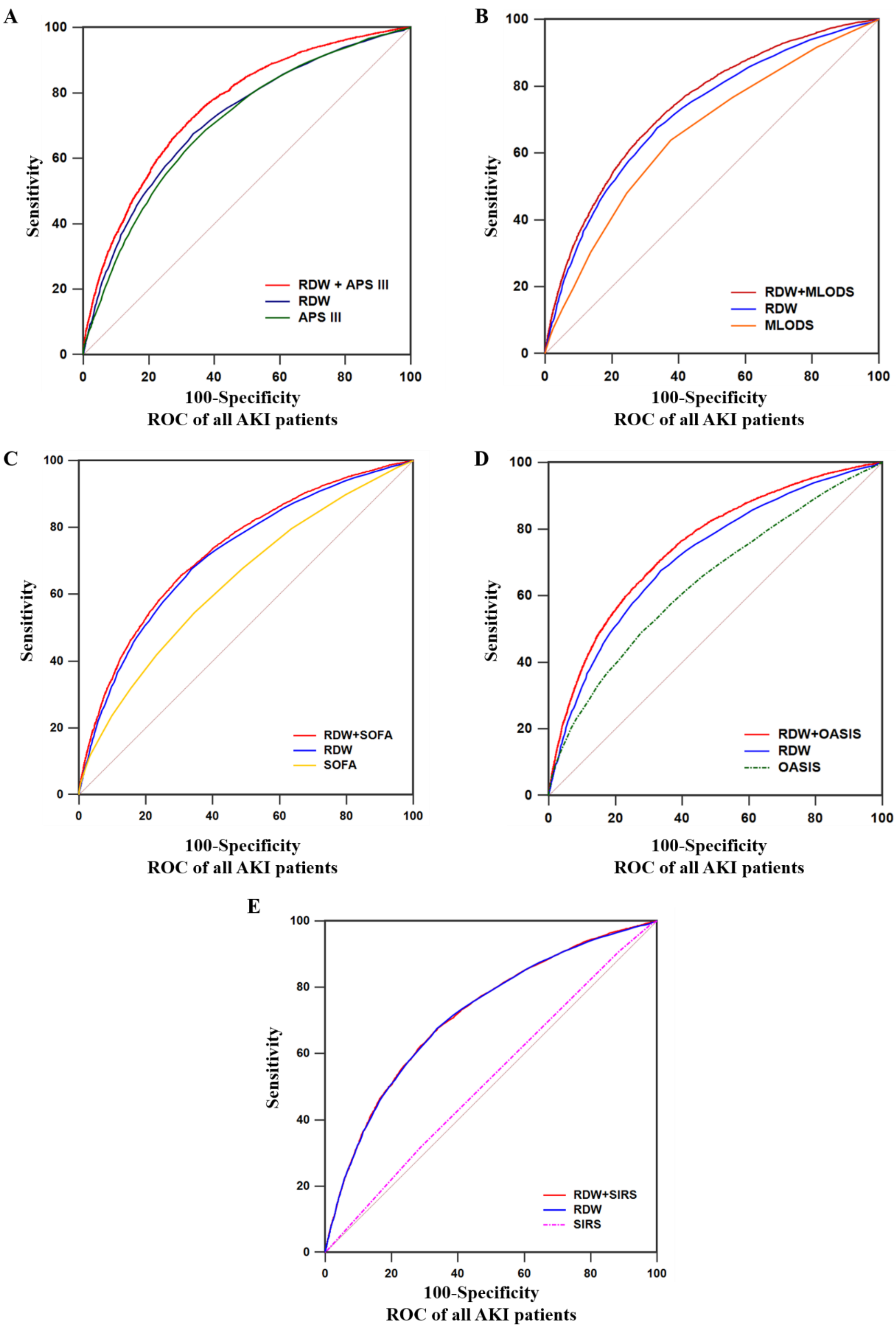
A
Sensitivity
100-Specificity
ROC of all AKI patients
RDW + APS III
RDW
APS III
B
Sensitivity
100-Specificity
ROC of all AKI patients
RDW+MLODS
RDW
MLODS
C
Sensitivity
100-Specificity
ROC of all AKI patients
RDW+SOFA
RDW
SOFA
D
Sensitivity
100-Specificity
ROC of all AKI patients
RDW+OASIS
RDW
OASIS
E
Sensitivity
100-Specificity
ROC of all AKI patients
RDW+SIRS
RDW
SIRS
